# Reviewed article: Amaral AB, Azeredo VMGO, Pereira LA, Rinaldi AEM. Knowledge and actions for the promotion and protection of breastfeeding by primary health care professionals in Uberlândia, 2022. Epidemiol Serv Saude. 2026:35;e20240245

**DOI:** 10.1590/S2237-96222026v35e20240245.b

**Published:** 2026-02-16

**Authors:** Carla Cristina Enes

**Affiliations:** 1 Pontifícia Universidade Católica de Campinas, Campinas, SP, Brazil

## First round comments

Prezados autores,

com base nos pareceres dos revisores, destacam-se três eixos principais de aprimoramento para os autores: a reformulação do título e da introdução; o detalhamento metodológico; e o fortalecimento da discussão e conclusão. Quanto ao título, ambos os pareceres recomendam torná-lo mais informativo e atrativo, sugerindo a inclusão da população e do foco no conhecimento, além de evitar o termo "estudo transversal".

Já a introdução deve justificar com mais clareza a relevância da experiência local de Uberlândia para um público nacional, além de apresentar de forma mais acessível conceitos e estratégias como a NBCAL, o SISVAN e a Estratégia Amamenta Brasil. O objetivo do estudo também deve ser reescrito para refletir a complexidade das variáveis analisadas, ampliando o escopo além das ações de promoção e proteção ao aleitamento materno.

Em relação à metodologia, os revisores apontam fragilidades que comprometem a reprodutibilidade e a interpretação dos achados. É fundamental esclarecer o universo amostral e os critérios de seleção das unidades e dos profissionais, além de justificar a escolha por perguntas abertas e descrever com mais precisão o processo de categorização das respostas. A apresentação dos resultados também deve ser reestruturada, alinhando texto e tabelas, evitando informações desnecessárias e reformulando expressões que dificultam a compreensão.

A discussão carece de maior densidade analítica e articulação com o estado da arte. Os autores devem contextualizar os achados à luz da literatura científica nacional e internacional, destacar contribuições originais do estudo e evitar repetições. A conclusão, deve superar o resumo dos achados e apresentar implicações práticas para políticas públicas e serviços de saúde, além de sugestões para futuras investigações.

Recommendation: Major revision.

